# Development of an *in vitro* platform for epithelial-stromal interactions: A basement membrane-containing scaffold from decellularized porcine bladders

**DOI:** 10.1016/j.mbplus.2025.100169

**Published:** 2025-02-22

**Authors:** J.A. Ramirez, S. Estrada, M.C. Harmsen, P.K. Sharma

**Affiliations:** a University of Groningen, University Medical Center Groningen, Department of Biomaterials and Biomedical Technology, Antonius Deusinglaan 1, 9713 AV Groningen, The Netherlands; b University of Groningen, University Medical Center Groningen, Department of Pathology and Medical Biology, Hanzeplein 1 (EA11), 9713 GZ Groningen, The Netherlands; c Tissue Engineering and Cells Therapy Group (GITTC), Cell Therapy and Biobank, Alma Mater Hospital of Antioquia, School of Medicine, University of Antioquia. Medellín, Colombia, Cra. 51a #62-42 Medellín, Colombia

**Keywords:** Basement membrane, Basal lamina, Decellularized extra cellular matrix, Scaffold cell culture

## Abstract

The first organized extracellular matrix that appears during mammalian embryogenesis is a basement membrane (BM), BM is present in all adult epithelia, endothelia, muscle, nerve and fat tissues. BM is a sub-micrometer thick compact lattice of macromolecules that is maintained by the adhered cells. Systems such as collagen gels, Matrigel® or synthetic polymeric scaffolds have been proposed to mimic the BM and to study the interactions between different cell types, but all lack a structured BM. Here we aimed to obtain and characterize a natural, thin basement membrane-containing scaffold from pig urinary bladders that are subjected to blunt dissection of layers and decellularization steps, preserving the near native BM with a few layers of underlying connective tissue to maintain its structural integrity. The scanning electron microscopy, confocal multiphoton microscopy and immunohistochemistry helped confirm the presence of the BM. A veil-like network composed of thin fibers was present on top of a course network, and glycosaminoglycans, collagen and basement membrane proteins were present. The scaffold’s ability to repopulation and basement membrane barrier function were further confirmed when HaCaT and MRC5 cells attached and remained respectively on the epithelial and mesenchymal side without any crossover. Cells remained viable till 2 weeks. This BM-containing scaffold allows to create *in vitro* models of epithelial-mesenchymal tissues through a structured basement membrane and investigate basement membrane dynamics. The basement membrane-containing scaffold was found to be isotropic under uniaxial tension with a failure strain of 0.25 allowing its use to investigate strain induced basement membrane dynamics.

## Highlights

• Existing basement membrane *in vitro* models lack a structured basement membrane.
• Our scaffold preserves the native tissue composition architecture, and mechanics.
• The scaffold is suitable for *in vitro* tissue modeling and basement membrane dynamics research.

## Introduction

An epithelium is a layer or layers of cells covering body surfaces such as skin, gastrointestinal track or genito-urinary track. Epithelial cells rest upon a thin layer of extracellular matrix (ECM) termed basement membrane (BM) [1], [2]. This compact layer has a thickness that, depending on the employed method of measurement and tissue type, can vary between 100 and 1,000 nm in most tissues, and is composed of intertwined networks of collagen type IV and laminin while proteoglycans such as perlecan and nidogen interconnect the network forming a dense sheet-like structure [3]. The BM has a higher density than the interstitial extracellular matrix. In fact, it acts as a barrier for cells and even macromolecules. As such, it is a molecular sieve. It separates epithelial and mesenchymal tissues, promotes epidermal/dermal attachment, drives cell polarization, and facilitates a controlled molecular exchange [4]. In mammals, it originates in the trilaminar embryonic disc, emerging as the first organized ECM, contributing importantly to development by local changes in its mechanical properties [5], [6]. Tensile forces sculpt the embryo during gastrulation guiding epiblast cells organization to form the trilaminar embryo. Later, forces at the tissue level drive neural tube closure and somitogenesis [7]. In mice embryos, growth is accompanied by BM perforations due the expression of matrix metalloproteinases (MMPs), which further enable the formation of the primitive streak, which defines the embryo’s bilateral symmetry and facilitates gastrulation initiation, highlighting the importance of BM remodeling in dynamic mechanical and biochemical scenarios [8].

Mechanical properties of the extracellular matrix together with external physical forces, controls the cell differentiation, proliferation, and tissue remodeling in healthy and diseased situations [9]. Even cellular homeostasis is dictated by matrix mechanics [10]. Nevertheless, our knowledge of the mechanical behavior of the epithelial BM and its turnover under mechanical strain remains poorly understood. One of the important reasons is the difficulty in isolating it while keeping its molecular structure and arrangement intact. Often *in vitro* BM models in the form of viscous gels such as Matrigel (Corning), Geltrex (Invitrogen), Cultrex (Trevigen), polymeric membranes or electrospun lattices are used, but they fail to accurately recapitulating the BM’s complex ultrastructure, topography, composition, and mechanical properties [11], [12]. Additionally, most of the known systems based on synthetic polymers do not allow BM remodeling and turnover by cells, a crucial aspect of ECM physiology. Therefore, the development of *in vitro* BM models that replicate its unique biomechanical and physiological characteristics is warranted.

To investigate BM remodeling, we anticipated that a natural BM would serve as the most appropriate starting point. Scaffolds can be obtained by decellularizing tissue e.g. by enzymatic, chemical and/or mechanical treatment. In general, efficient decellularization demands the use of detergents such as Triton X-100 (non-ionic), sodium dodecyl-sulfate (SDS) or sodium deoxycholate (SDC) (ionic), 3-[(3-cholamidopropyl) dimethylammonio]-1-propanesulfonate (CHAPS, zwitterionic). The choice of detergent, concentration, combination, and exposure time often depends on the specific tissue source. Detergent use is a necessary evil because they are most effective in dissolving and removal of cells, yet may disrupt the ECM and thus the BM structure [13]. Research in this field is mainly focused on clinical applications [14], [15], and for some, the employed tissues may conserve a BM together with a millimetric wall of stromal ECM, which is the case of dermal ECM for instance (Alloderm^TM^, Strattice®, Cortiva® Allograft Dermis), or the porcine small intestine submucosa (SIS) from RTI surgical [16], [17]. Decellularized ECM products such as SIS have been employed for *in vitro* cell culture for a variety of cell types [18]. While these materials has been reported to enhance cell survival [19], and have been utilized to mimic tissues ranging from cardiovascular to bone [20], [21], as well as to develop co-culture [22], [23], tumoral [24], [25] and infection models [26] including studies incorporating mechanical stimulation[27], [28], the volume of literature exploring BM characterization or the use of these models to study BM biology and dynamics remains limited, despite their potential and physiological relevance.

Motivated by these considerations, in this study we sought to establish a basement membrane-containing scaffold derived from the porcine urinary bladder mucosa. Through a comparative evaluation of three decellularization protocols, we selected a mild and efficient method to preserve the essential components and ultrastructure of the extracellular matrix with minimal alterations to the basement membrane. This scaffold, characterized by its accessibility and substantial surface area, serves as an *in vitro* platform for investigating epithelial-mesenchymal interactions and basement membrane dynamics.

## Results

### Overall ECM integrity and composition

**Fig. 1:**
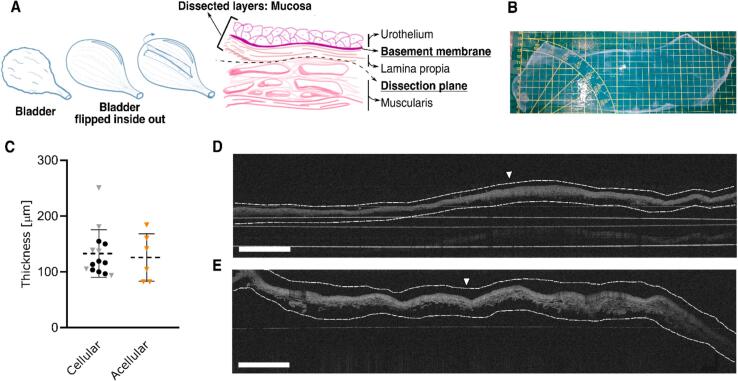
**Dissection of procine urinary bladder to isolate the mucosa A)** schematic of the proceadure for obtaining the mucosa with a final appereance depicted in **B).** Measured thickness before and after decellularization is plotted in **C)** and cross sectional views are shown in **D)** and **E)** before and after decellularization respectively (highlighted by dashed lines). 14 mucosas were employed and from those, 6 (represented by triangles) were taken for decellularazation. On each of the samples, three randon fields were scanned and at least 10 points per field were measure**.** Arrowheads indicate the basement membrane surface. Scale bars are 1 mm.

We established a reproducible procedure to consistently and reliably obtain thin porcine urinary mucosae (coefficient of variation in thickness ≈ 30 %). The membrane was translucent (Fig. 1B) and easy to handle. Isolated mucosae had an area of approximately 300 cm^2^ (Fig. 1B) and a thickness of 133 ± 42 μm (Fig. 1C). The optical coherence tomography (OCT) scans clearly distinguished both surfaces of the sample (Fig. 1D). One surface had a smooth and continuous appearance, most likely the BM surface (indicated with an arrowhead), while the other had a rough texture with protruding fibers, probably the stromal ECM, as a result from the dissection process. After decellularization the thickness did not change (126 ± 43 μm, Fig. 1C) and the smooth and continuous surface remained as shown in the OCT scans (Fig. 1E, arrowhead indicating the smooth surface).

**Fig. 2:**
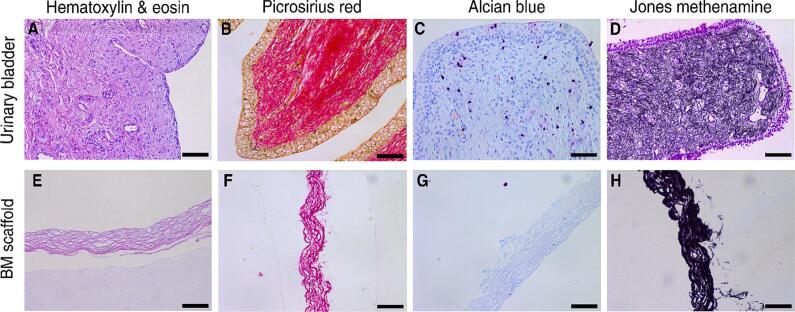
**Histological characterization of the basement membrane-containing scaffold compared to the porcine urinary bladder cross sections. A)** and **E)** Hematoxylin and Eosin**, B)** and **F)** picrosirius red staining collagen network**, C)** and **G)** Alcian blue staining GAGs and **D)** and **H)** Jones methenamine silver staining the BM. All scale bars are 100 μm.

After 3 h decellularization, there were no discernible nuclei in the 1 % SDS group (Fig. 2E and Supplementary Fig. 2C), in contrast to the other groups, DM water and 1 % v/v Triton X-100, where there were still visible nuclei after 12 h, without visible ECM damage (Supplementary Fig. 2); hereon, we worked only with the 1 % SDS, 3 h group. Collagen fibers (Fig. 2B and 2F) and glycosaminoglycans (Fig. 2C and 2G) were conserved after decellularization as it is shown by the Picrosirius red and Alcian blue staining respectively. Jones methenamine, routinely used to visualize glomerular BM, stained positive not only the BM but the complete *lamina propia* and was conserved after decellularization (Fig. 2D and 2H).

### The main constituents of the BM are conserved in decellularized scaffolds

**Fig. 3:**
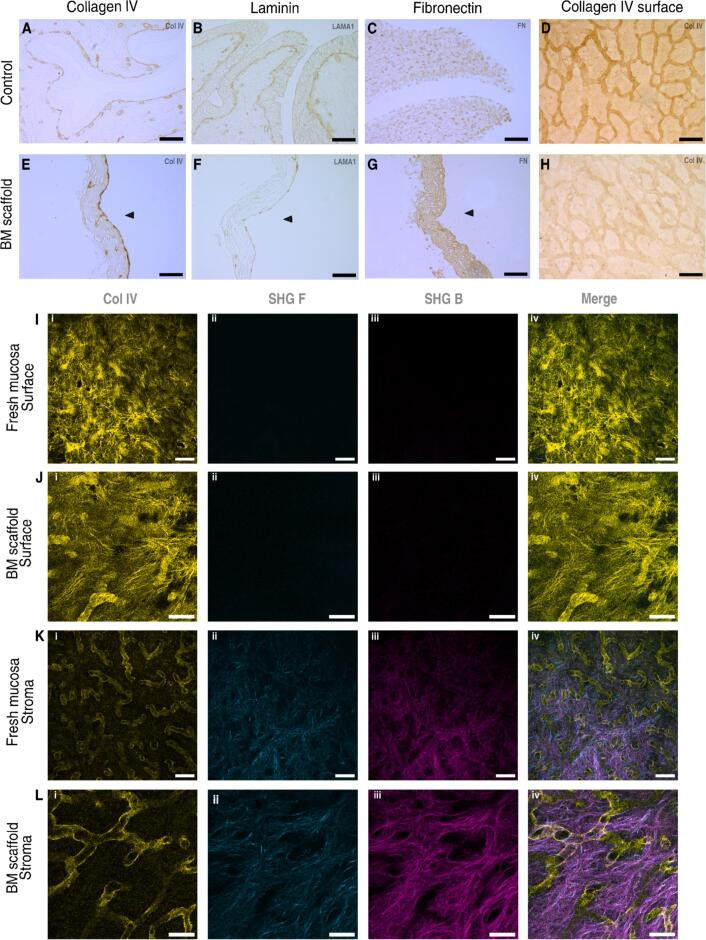
**Relevant basement membrane components of the scaffold.** Immunohistochemical (IHC) cross sections for **A)** and **E)** collagen type IV, **B)** and **F)** laminin α-1**, C)** and **G)** fibronectin-1 and surface for collagen IV **D)** and **H)** of intact bladder or mucosa as control (A, B and C, or D respectively) and basement membrane-containing scaffold respectively. Scanning confocal microscopy for collagen IV and second harmonics generation signals forward and backward (SHG F and SHG B)) for the mucosa’s surface **I)** and stroma **K)** compared to the basement membrane-containing scaffold surface **J)** and stroma **L),** the images correspond to a representative confocal section. Scale bars for IHC are 100 μm. Arrowheads indicate the basement membrane surface. Scale bars for confocal images are 50 μm.

The decellularization procedure had subtle impact on both major BM protein network, collagen type IV and laminin. A continuous sheet of collagen IV and laminin network was clearly visible surrounding the capillaries and separating the transitional epithelium from the stromal ECM in the intact urinary bladder denoting the presence of the basement membrane (Fig. 3A and 3B). Post decellularization, the basement membrane remained continuous on the surface of the scaffold and surrounding capillaries (Fig. 3D and 3E), as shown by the collagen IV and laminin networks. Stained collagen IV intensity, post decellularization, seems comparable to the intact bladder on the epithelial surface side, the laminin staining whereas, shows a reduced intensity (Fig. 3E and 3F). Fibronectin, an important ECM glycoprotein implicated in mechanotransduction and closely associated with the basement membrane [29], [30], was homogenously distributed within the tissue before and after decellularization (Fig. 3C and 3G).

We further confirmed the presence of collagen IV all over the sample, presenting a stronger signal coming from its interconnected structure (Fig. 3D and 3H). Confocal microscopy together with SHG, which allows the detection of fibrous collagens only (not collagen IV), as spatial context confirmed that the collagen IV layer covered the surface both in the control (mucosae without decellularization treatment) and the BM-containing scaffold and it was detected above the SHG signal (Fig. 3I and 3 J respectively). Deeper in the z-direction, the collagen IV signal did not disappear completely but remained as structures similar to those seen by immunohistochemistry, and the fibrillar collagens started to appear as the predominant stromal ECM (Fig. 3K and 3L). The interconnected structures were seen as tubular formations within the stromal ECM with a diameter around 25 μm, most likely blood or lymphatic capillaries (Supplementary Videos S1 and S2).

### A veil-like layer covers the collagen fibers

**Fig. 4:**
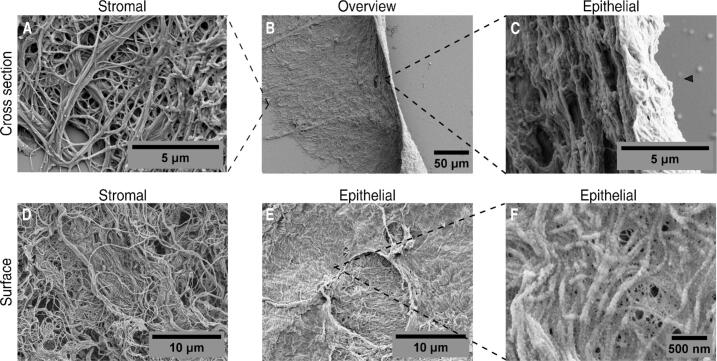
**Electron scanning micrographs of the basement membrane scaffolds A)** amplified cross-sectional view of the outermost stromal region, **B)** cross sectional overview, **C)** amplified cross-sectional view of the basement membrane surface, arrowhead indicates the basement membrane surface. **D)** Stromal ECM surface, **E)** epithelial**/**basement membrane surface and **F)** magnification of the basement membrane surface revealing nanometric pores and fibers.

We imaged the micro and nano architecture of the decellularized membranes with scanning electron microscopy and saw collagen fibers with the characteristic D-period in a loose and in a random-oriented fashion with a tortuous topography both in the cross section and surface, which is a typical characteristics of the stromal surface (Fig. 4A and 4D). In contrast, the epithelial surface had a compact and flat appearance characterized by a high density of collagen IV fibers (Fig. 4C, 4E in combination with 3E and 3 J). Notably, the layers closer to the epithelial surface seemed tightly stacked (Fig. 4C) in comparison to the most stromal layers (Fig. 4A). Throughout the epithelial surface, there were discontinuities resembling pores to the stromal space (Fig. 4E). A thin, compact layer, likely the basement membrane, appeared to be in direct contact with the underlaying fibrous structure in a veil-like manner (Fig. 4F), with nanometric pores within a densely packed mesh composed of thin fibers (fiber thickness of veil-like structure and underlaying collagenous fiber: 10 ± 2 nm and 64 ± 12 nm respectively).

### Basement membrane-containing scaffolds are isotropic and slightly stiffer after decellularization

**Fig. 5:**
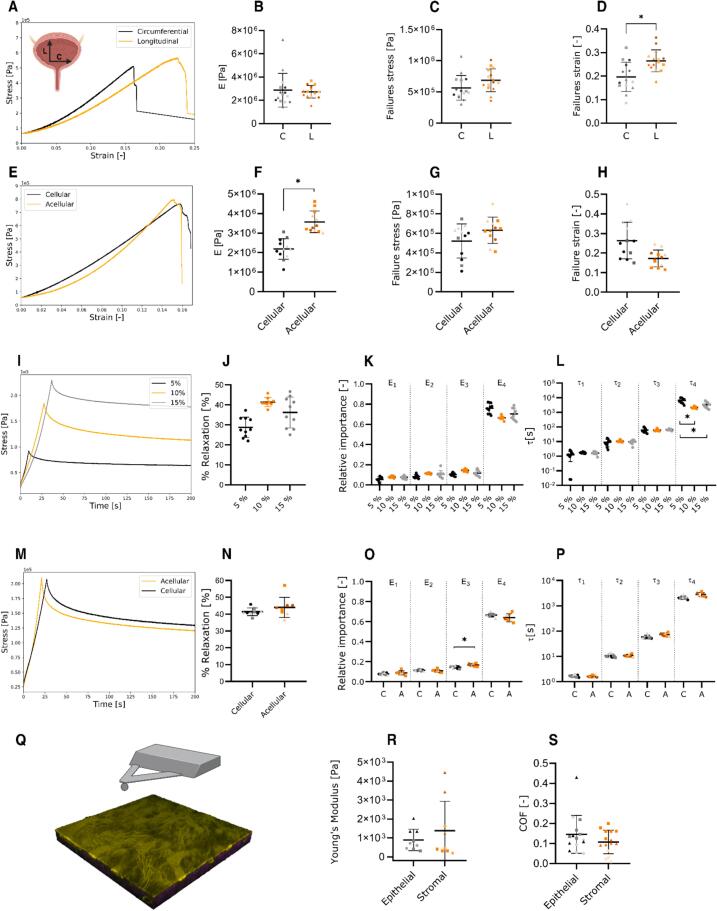
**Mechanical characterization of the fresh membrane and basement membrane scaffold A)** Representative strain–stress plots, **B)** Young’s modulus, **C)** failure stress, and **D)** failure strain in the circumferential and longitudinal direction. **E)** Representative strain–stress plots, **F)** Young’s modulus, **G)** failure stress, and **H)** failure strain comparing before and after decellularization. **E)** Representative stress relaxation plots, **J)** percentage of relaxation**, K)** relative importance and **L)** relaxation constants of the Maxwell elements at 5 %, 10 % and 15 % strain. **M)** Representative stress relaxation plots, **N)** percentage of relaxation**, O)** relative importance and **P)** relaxation constants of the Maxwell elements before and after decellularization at 10 % constant strain. **Q)** Schematic representation of the nano-compression test with AFM, **R)** Young’s modulus at nano-compression. Each individual point in the plots represents the average value of a replicate, these individual values are grouped by shape (■▲●♦) representing each independent experiment (N = 4 for B, C and D, N = 3 otherwise).

The mucosae showed an isotropic behavior with comparable Young’s moduli under tension in circumferential and longitudinal directions respectively: 2.9 ± 1 MPa and 2.7 ± 0.4 MPa, Fig. 5B) and similar failure stresses (0.56 ± 0.12 MPa and 0.67 ± 0.12 MPa, Fig. 5C). Yet, failure strains were different (0.20 ± 0.05 and 0.26 ± 0.03, Fig. 5D). After decellularization, the Young’s modulus increased up to 3.5 ± 0.3 MPa (Fig. 5F) and failure stress and strain did not vary significantly (Fig. 5G and 5H respectively) with the average values of 0.63 ± 0.05 MPa and 0.17 ± 0.03 respectively. The form of the stress–strain curve is predominantly linear, which differs from the typical j-shaped curve of most biological tissues, including the whole bladder wall (not tested here, but can be found in Jokandan et al. 2018), and a brittle failure with little to no plastic deformation.

In order to study the viscoelastic behavior the samples were held at constant strains of 0.05, 0.1 and 0.15 (well within the failure strain) for 200 s. Comparable stress relaxations of 29 ± 5 %, 41 ± 0.8 % and 35 ± 8 % were observed respectively. The Maxwell components analysis yielded 4 elements which were required to fit the measured relaxation dtat in all the cases, where the fourth one was the most relevant with a relative importance, ${\mathrm{RI}}_{4}$ of 70 % and $\tau_{4}$ ranging from 2,000 s to 6,000 s. These results imply that the material has a viscoelastic solid behavior up to 15 % strain and is mainly isotropic under tension. Furthermore, decellularization seems to have neither changed the %stress relaxation (Fig. 5N) nor the Maxwell parameters (Fig. O, P).

Under nano-compression, AFM showed that the Young’s modulus of the epithelial side was of the order of 1 kPa (0.9 ± 0.6 kPa), comparable to 1.3 ± 1.3 kPa from the stromal side. Also, the measured coefficients of friction of the epithelial and stromal surfaces 0.14 ± 0.03 and 0.09 ± 0.06 respectively, were not different either.

### The basement membrane-containing scaffold was receptive to epithelial and mesenchymal cells

**Fig. 6:**
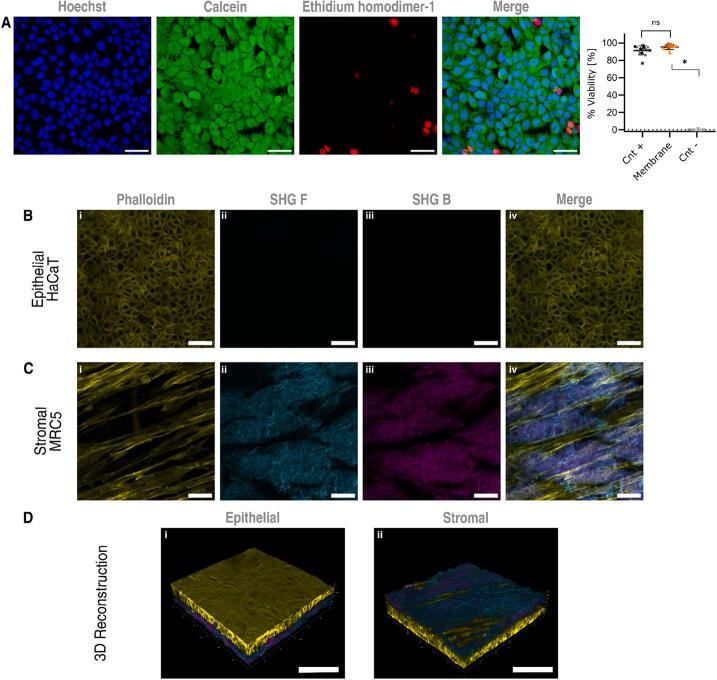
**Cells health and coculture on the basement membrane scaffold A)** LIVE/DEAD viability of HaCaT cells seeded on the epithelial side (N = 3), and coculture of **B)** HaCaT cells on the epithelial side and **C)** MRC5 lung fibroblast on the stromal side of the basement membrane-containing scaffold surrounded by collagen fibers, scale bars are 50 μm. **D)** 3D view of the coculture from top and bottom isometric views, scale bars are 100 μm.

HaCaT cells were seeded on the epithelial side of the scaffold and the viability did not differ from control cells cultured on plastic after 24 h i.e. 95 ± 2 % vs 91 ± 3 % respectively (Fig. 6A). Furthermore, we seeded fibroblasts to the stromal side and kept the coculture for 14 days to assess the long term separation of cellular layers of these epithelial-mesenchymal constructs (Fig. 6D). HaCaT cells adhered and formed a confluent monolayer on the epithelial i.e. the basement membrane side, while MRC5 cells adhered to the stromal ECM in a more scattered fashion, retaining their characteristic spindle-like morphology (Fig. 6C). Some fibroblasts migrated within the stromal ECM toward the epithelial-stromal interface (data not shown), but could not penetrate it. Similarly, HaCaT cells did not cross into the stromal ECM, implying that the scaffold’s barrier function was intact. The collagenous structure on the stromal ECM as seen by SHG showed a change in the fibers pattern, after coculturing for 14 days it appeared less organized, containing thinner and shorter fibers and in a more diffuse manner (Fig. 6C ii and iii) as compared to the initial architecture where fibers seemed larger and more structured, arranged around capillaries (Fig. 3K ii and iii or 3L ii and iii) strongly suggesting ECM remodeling.

## Discussion

We developed and characterized a basement membrane (BM)-containing scaffold from porcine bladder wall through blunt dissection and decellularization, with consistent thickness. This benefits the reproducibility of the mechanical performance and homogeneity of cell volumetric density during recellularization. The scaffold biochemical, mechanical and ultrastructural properties studied were comparable with those of the intact, cellular bladder mucosa and it kept the natural barrier function between seeded epithelial and mesenchymal cells for at least 2 weeks. Isolation of BM is challenging since they are generally firmly attached to the stromal ECM and are very thin and difficult to handle on their own. Interestingly, other researchers have successfully prepared functional membranes, however, often compromising epithelial surface continuity, cell infiltration, or experiencing collagen denaturation [32], [33], [34]. Building upon this existing knowledge, and employing a simple and short decellularization procedure, we obtained a reinforced, continuous and smooth BM-containing scaffold with a thin (∼120 μm) stromal tissue layer providing mechanical stability and robustness for ease of handling. The preservation of the basement membrane’s structural integrity renders it suitable for biomechanical and cell biological studies.

In our study we have retained the main BM proteins and GAGs with some lowering in laminin staining; moreover, the ultrastructure and barrier function of BM seems to be preserved. Epithelial cells seeded over the basement membrane side of the scaffold did not migrate into the stromal ECM, and mesenchymal cells were found on several layers within the stromal ECM, some even reaching up to the epithelial interface, but never trespassing the interface, which allows for epithelial-mesenchymal interactions. Furthermore, neither epithelial nor mesenchymal cells showed any morphological changes, indicating that our scaffold is suitable for cells re-seeding and co-cultures.

The presence and distribution of collagen IV within the BM was confirmed by combining SEM and confocal-multiphoton imaging. The epithelial basement membrane was efficiently distinguished from the underlaying vascular network, which also possesses a BM of their own. At the ultrastructural level, the BM has been reported to have a felt-like topography, consisting of intertwined fine fibers and pores on the nanoscale [11]. Our results revealed a dense, veil-like sheet composed of nanometric fibers overlying a network of thicker fibers. Incidentally, these nanofibers (shown in white in Fig. 4F) are clearly visible within the black spaces. When taken together these observations with the confocal immunostaining, this veil-like sheet corresponds to the collagen IV network. It is clear from our results that the stromal ECM underlaying the basement membrane looks compact compare with the stromal surface, it has not been studied how this density gradient may affect the biomechanics and mechanotransduction of the epithelium though.

The whole urinary bladder wall is reported to be mechanically anisotropic in the longitudinal and circumferential directions, but as our results suggest, the basement membrane-containing scaffold was isotropic. Rosario and collaborators, reported very similar values for the stiffness of a urinary bladder matrix [34] in the order of 2–3 MP, also isotropic in the longitudinal and circumferential orientation. The stress–strain curves of our basement membrane-containing scaffolds were closer to linear than to the typical j-shaped curves of soft biological specimens, we expect that this happens because of the lack of elastin in the scaffold (supplementary Fig. 5), since the elastin in the bladder wall is located just between the *lamina propria* and the detrusor muscle [35], [36]. Our results show that the stiffness increased after decellularization. Some studies claim similar results or no contribution of cells to overall tissue stiffness [11], [37], [38], but some report, unlike our data, a positive contribution by cells; this heterogeneity has been proposed to be a tissue specific response, however, the underlying causes of this phenomenon remains unclear [39]. The nano-compression tests on the other hand, yielded a Young’s modulus 3 orders of magnitude smaller that the tensile modulus, which is in accordance with other reports [40]. Nevertheless, absolute stiffness or Young’s modulus values has been shown to vary considerably among tissues, developmental stage, disease and techniques employed [41]. We expected the stiffnesses and coefficients of friction of each side to be different, due to their intrinsic architectural difference. However, our current methodology did not reveal any appreciable differences.

During pregnancy, the abdominal skin has shown to, over a period of months, stretch to 40 %, which, according to simulations, can only be possible if the process is accompanied by tissue remodeling and growth [42]. A similar situation may apply during growth and development, bodybuilding, weight gain etc. However, little is known about the tensile properties of the BM, like the extent to which BM itself can be stretched, when loaded at once (i.e. not having time to remodel over the span of months). Epithelia like in the lung alveoli are estimated to stretched from 4 to 12 %, complete bladder walls on the other hand has been reported to sustain a 43.5 % circumferential and 35.8 % longitudinal strain [31]. The BM-containing scaffold isolated from bladder by us showed a brittle failure with an ultimate deformation of around 25 % (i.e. failure strain, *ε_f_*, of 0.25). The reason for the BM-containing scaffold to have half the failure strain as compared to whole bladder is perhaps because of the lack of urothelial folds in the scaffold. There are some other models available e.g. Li and collaborators implemented a spheroid-based system to study the nonlinear biomechanics of *in vitro* deposited 2.5 μm BMs by inflation and deflation. In that research, after detaching the cells, they stretched the spheroid by 30 % through application of a pressure of 40 kPa without causing evident damage to the spheroid [43]. Thus, we can safely assume that the BM in our BM-containing scaffold remains intact till its failure deformation of 25 %.

Biological tissues and their derivates usually exhibit non-elastic properties, our scaffold for instance, behaves as a viscoelastic solid with around 40 % stress relaxation in 200 s. Interestingly, 4 Maxwell elements with approximately the same relaxation constants and elements with similar relative importances were required to fit the stress relaxation of cellular and acellular tissue. This implies that with the technique employed, presence of cells did not alter the viscoelasticity of the urothelium. Out of the 4 Maxwell elements, the fourth one contributes to around 70 % and has a relaxation time characteristic of solids [44], due to its abundance, this fourth element could correspond to the collagen fibers; and most likely the first element, with the shortest relaxation time, represents free water. The other two intermediate elements could represent adsorbed water, other ECM structures such as fibronectin or the BM. Due to the nature of the generalized Maxwell model, it is not possible to relate each element to a specific constituent of the scaffold.

It has been shown that the basement membrane is not solely produced by epithelial cells [45], [46]. For instance, in drosophila, muscle and fat tissues located remotely from the basement membrane’s final anatomical position, has been shown to play a substantial role in its formation and repair [47], thereby, a platform for BM *in vitro* research must allow epithelial to mesenchymal communication. In this way, our basement membrane-containing scaffold facilitated the coculture of epithelial and mesenchymal cells, and the construct could be maintained for up to 2 weeks in standard culture conditions (longer duration needs future testing). Both cell populations conserved their physiological location, and there were subtle signs of ECM remodeling that deserve further investigation. Techniques like metabolic labeling [48], employing fluorescent tagged proteins [49], analyzing ECM rearrangement and changes in mechanical properties can be used to remodeling. Our BM-containing scaffold enables studies on transmembrane diffusion, cell migration/invasion across epithelial barriers, and their effects on tissue remodeling and homeostasis. Furthermore, its ability to stretch up to 25 % allows application of diverse mechanical stimuli for mechanobiology research. Moreover, it can be potentially employed to create more complex and physiologically relevant organotypic *in vitro* models such as skin, cornea, renal glomeruli, mammary ducts, endothelia, brain-blood barrier or blood-placental barrier.

## Methods

### Mucosa preparation

Porcine urinary bladders of female animals between 4 and 8 months old were obtained from a local slaughterhouse (Kroon Vlees b.v., Groningen, The Netherlands) and processed within 3 h after collection. Connective tissue and fat were removed, the bladder was flipped inside-out and the mucosa, i.e. urothelium with undelaying *lamina propia*, was blunt-dissected following the dissection plane closest to the urothelial surface, while keeping the tissue in PBS at room temperature (Fig. 1A). For mechanical testing, mucosae were preserved in PBS and used the same day. All other samples were stored in demineralized (DM) water and frozen at −20 °C.

### Optical coherence tomography

Optical coherence tomography (OCT, Thorlabs Ganymede II, Newton, USA) was employed to quantify the thickness of the mucosae and scaffolds. The samples were placed in petri dishes containing PBS and taken to the OCT system, which was configured with a refractive index n = 1.33, corresponding to water at 20 °C. 2D images of cross sections were scanned over an area of 10 mm X 2.9 mm. At least 10 thickness measurements were obtained per section and 3 random sections were scanned per sample.

### Decellularization

Three different decellularization methods were investigated to obtain the basement membrane-containing scaffolds. Mucosae were thawed and washed once with DM Water, afterwards, each sample was put in 40 ml of any of the following media at 4 °C in constant agitation: DM Water, 1 % v/v Triton X-100 solution (Sigma Aldrich, San Luis, USA) or 1 % w/v sodium dodecyl sulfate (SDS, Sigma Aldrich) solution. Small samples of 4 cm^2^ were taken every 3 h up to 12 h to assess the presence of nuclei by hematoxylin & eosin staining. Then, the samples were washed 10 times with DM water to remove traces of the detergent and finally stored in PBS.

For cell culturing, basement membranes-containing scaffolds were prepared after 3 h treatment with 1 % w/v SDS, and afterwards handled under sterile conditions completing 10 washes with sterile DM water and finally stored in PBS containing 1 % penicillin/streptomycin (Gibco, New York, USA) at 4 °C.

### Histology, immunohistochemistry and immunofluorescence

Histological analysis was performed to assess the extent of decellularization, compare the morphology of the basement membrane-containing scaffolds to the native urinary bladder and investigate the content of crucial biochemical constituents of the BM and underlying stromal ECM. Fresh bladders and basement membrane-containing scaffolds samples were fixed in 4 % paraformaldehyde in PBS (Klinipath, Duiven, The Nerherlands) for 72 h at room temperature, dehydrated and paraffin embedded. Sections of 4 μm were obtained (Epredia HM 355S, Kalamazoo, USA), cleared with xylene (Fresenius, Bad Homburg, Germany) and rehydrated in graded ethanol series (Fresenius) to be subsequently stained with hematoxylin and eosin, picrosirius red, Alcian blue or Jones methenamine silver.

For immunohistochemistry, sections were processed alike; after rehydration, antigen retrieval was performed at 85 °C overnight in 0.1 mM Tris HCl (Millipore, Burlington, USA) pH 9. Blocking solution (1 % BSA, 5 % serum from the secondary antibody host animal in PBS) was added for 30 min, then, primary antibody diluted in blocking solution was incubated for 90 min and washed 3 times with PBS, followed by a 10 min incubation in 1 % hydrogen peroxide (Sigma Aldrich). After washing, secondary antibody conjugated with horseradish peroxidase (HRP) and diluted 1:100 in PBS containing 2 % swan serum was incubated for 30 min and washed 3 times with PBS, then DAB (Sigma Aldrich, cat: D4293) was added and incubated for 10 min and finally, sections were washed 3 times with DM water and mounted in aqueous mounting media.

To visualize the distribution of BM associated collagen IV on fresh mucosae or scaffolds, non-fixed, fully hydrated samples of 8 mm diameter were cut with a punch and placed on a glass slide with the BM facing upwards and stained with the primary antibody as for immunohistochemistry. Finally, the sample was stained with secondary fluorescent antibody diluted 1:500 in PBS containing 2 % swan serum for 30 min, washed and mounted with PBS for imaging with a confocal-multiphoton microscopy set up, employing confocal microscopy for labeled collagen IV and second harmonics generation (SHG) to identify fibrillar collagens, since collagen IV does not produce SHG signal. As control, a fresh, non-decellularized sample was stained with previous permeabilization with Triton X-100 0.5 % for 10 min. Validation negative staining for collagen IV antibody on cartilage is reported in supplementary Fig. 3.

Primary antibodies employed were goat anti-collagen type IV (Southern Biotech, Birmingham, USA, cat: 1340–01, 1:25), rabbit anti-laminin α1 (Sigma Aldrich, cat: L-9393, 1:25) and rabbit anti-fibronectin 1 (abcam, Cambridge, UK, cat: ab2413, 1:200). All antibodies cross reacted with porcine tissue, tested on porcine kidney. Secondary antibodies were goat anti-rabbit HRP (Dako, Santa Clara, USA, cat: P0448, 1:100), rabbit anti-goat HRP (Dako, cat: P0449, 1:100) and Mouse anti-goat TRITC (Jackson ImmunoResearch, Baltimore, USA, cat: 203–025-108, 1:500).

### Scanning electron microscopy

ECM architecture was investigated by means of electron scanning microscopy using two approaches, thick cross sections and surfaces of basement membrane and stromal tissue. For cross sections, 50 μm paraffin sections were prepared in the microtome (Epredia HM 355S), picked up onto glass cover slips, rehydrated in series of 100 %, 96 % and 70 % and air-dried.

For surfaces, bulk samples were fixed for 2 h in paraformaldehyde 4 % and washed twice, followed by a secondary fixation in microscopy grade glutaraldehyde 3 % (Merck, Darmstadt, Germany) and washed 5 times. Dehydration of the samples was achieved with grade series of *tert*-butanol (Sigma Aldrich, cat: 471712), 30 %, 50 %, 70 %, 85 % for 10 min each and 90 %, 95 %, 100 % for 3 min each. Samples were immediately frozen at −20 °C for at least 1 h and then freeze dried for 2–4 h.

Dry samples were sputter-coated (Leica microsystems, Wetzlar, Germany, EM ACE600 high vacuum sputter) with a 6–8 nm Chromium layer and then imaged in the electron microscope (Zeiss Supra 55, Oberkochen, Germany).

## Characterization of tensile mechanical properties

**Fig. 7:**
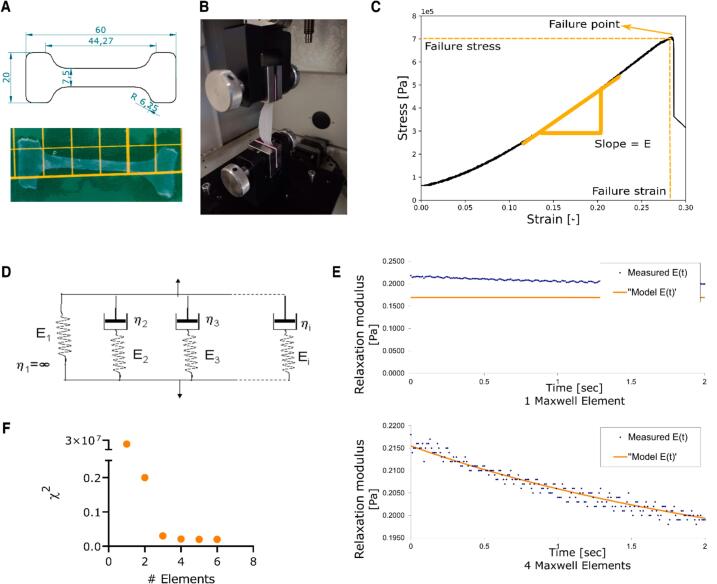
**Mechanical characterization methodology. A)** Dog bone shaped samples were cut out of the mucosa or basement membrane-containing scaffold and used for the tensile test. **B)** UMT setup for tensile tests**. C)** Representative strain–stress plot exemplifying the failure point, failure stress, failure strain and Young’s modulus as the slope of the linear region. **D)** Schematic representation of the generalized Maxwell model and **E)** representative relaxation plot fitting one Maxwell element (up) and four Maxwell elements (bottom). **F)** Decrease in **χ**^2^, representing the goodness-of-fit, for the generalized Maxwell model with increasing number of Maxwell elements.

Fresh mucosae (cellular) and basement membrane-containing scaffolds (acellular) were tested under tension in a universal mechanical tester (UMT-3, Bruker Inc, Billerica, USA) using a 10 N load cell (DFM-1–1242, Res 0.5 mN). Samples were cut into a dog-bone shape as shown in Fig. 7A and held with two pieces of foam paper on both sides and a drop of acrylic glue to be later mounted on the UMT. Blotting paper soaked in PBS was gently placed behind the sample to prevent it from drying (Fig. 7B). An initial preload of 40 mN was applied followed by a constant deformation speed of 150 μm/s, i.e. a strain rate of 0.34 /s, either until failure to determine the Young’s Modulus, failure stress, and failure strain (as exemplified in Fig. 7C), or up to 5 %, 10 % or 15 % strain kept constant for 200 s to determine the stress relaxation. Isotropy was tested on orthogonal samples, oriented circumferentially and longitudinally, and the effect of decellularization over the stress relaxation, Young’s modulus and failure was studied.

Young’s modulus was determined as the slope of the fitted linear regression of the stress–strain curve between 40 % and 60 % of the failure point. Stress relaxation in % was calculated as $100*\left(E\left(t_{0}\right)-E\left(t_{200}\right)\right)/E\left(t_{0}\right),$ where $t_{0}$ is the time when the constant strain is reached and $t_{200}=\;t_{0}+200\;s$. Engineering stress/strain values are reported, true values are reported in supplementary Fig. 4.

Young’s modulus, failure stress and strain were analyzed using a self-compiled Python program, and stress relaxation data was fitted with the generalized Maxwell model (equation (1), Fig. 7D and 7E) with a Matlab (MathWorks Inc, Natick, USA) script to describe $E\left(t\right)$ (equation (1), and determine the Maxwell elements $E_{i}$, their relaxation time constants $\tau_{i}$ (equation (2) and relative importance ${\mathrm{RI}}_{i}$ (equation (3). The minimum number *n* of Maxwell elements required to fit the experimental data was determined by looking at the drop in χ^2^ as a function of each additional Maxwell element (Fig. 7F and Supplementary Fig. 1).

(1) $$E\left(t\right)=E_{1}e^{-t/\tau_{1}}+E_{2}e^{-t/\tau_{2}}+\cdots +E_{i}e^{-\frac{t}{\tau_{i}}}\cdots .+E_{n}e^{-t/\tau_{n}}$$

Where:

(2) $$\tau_{i}=\frac{\eta_{i}}{E_{i}}$$

(3) $${\mathrm{RI}}_{i}=100*\frac{E_{i}}{\sum_{i=1}^{n}E_{i}}$$

### Atomic force microscopy

Friction force was measured as reported previously [50], with an AFM (Nanoscope IV Dimension tm 3100) equipped with a Dimension Hybrid XYZ SPM scanner head (Veeco, New York, USA) in contact mode employing a SAA-SPH-10UM tip (Bruker Inc, nominal constant 0.25 N/m) with a radius of curvature (ROC) of 10.25 μm. For this, the sample was mounted on a petri dish whose surface was covered with double-sided tape, kept in place with a metallic ring and submerged in PBS throughout the experiment. The torsional stiffness $K_{t}$ of the used tip was between 2 to 6 × 10^-10^ Nm/rad. The deflection sensitivity α of the tip was recorded at a constant compliance with bare glass in PBS to calculate the normal force $F_{n}$ applied using:

(4) $$F_{n}=\Delta V_{n}\propto K_{n}$$

where ${\Delta V}_{n}$ is the voltage output from the AFM photodiode due to normal deflection of the tip. The torsional stiffness and geometrical parameters of the probe were used to calculate the friction force $F_{f}$ according to:

(5) $$F_{f}=\Delta V_{l}K_{t}2\delta d+t/2$$

where t is the thickness of the cantilever, δ is the torsional detector sensitivity of the AFM and $\Delta V_{l}$ corresponds to the voltage output from the AFM photodiode due to lateral deflection of the probe. Lateral deflection was observed at a scanning angle of 90 degrees over a scan area of 5 × 5 μm^2^ and a scanning frequency of 1 Hz. The tip was incrementally loaded and unloaded up to a normal force of 60 nN. At each normal force, 16 friction loops were recorded to yield the average friction force.

The Young’s modulus under nano-compression was measured with the Catalyst AFM (Bruker Inc) coupled with a Leica DMI 4000B fluorescence microscope in contact mode employing a SAA-SPH-10UM tip (Bruker InC, nominal constant 0.22 N/m, ROC = 10.25 μm). The sample was placed on a glass slide and kept in place with a metal ring while submerged in PBS throughout the experiment, 3 random fields on each side of the samples were scanned and 30 force curves were recorded on each field (270 force curves per side in total), at a frequency of 1 Hz, tip velocity of 8 μm/s and a trigger threshold of 5 nN. The data analysis was performed in the NanoScope Analysis V1.8 with a Hertzian model using a Poisson’s ratio of 0.45.

### Cell culture

Epithelial (HaCaT, immortalized human keratinocytes) and stromal (MRC5, normal human lung fibroblasts) cell lines were cultured in DMEM HG (Gibco) high glucose supplemented with 10 % FBS (SERANA, 1 % L-glutamine (Lonza, Basel, Switzerland) and 1 % penicillin/streptomycin (Gibco) in standard culture conditions (37 °C and 5 % CO_2_ with saturating humidity).

### Viability

For determining the viability, HaCaT cells were seeded at a density of 60 x 10^3^ cells/cm^2^ on a basement membrane-containing scaffold mounted on a CellCrown^TM^ 12NX (Scaffdex, Tampere, Finland) holder system or 12 well plates for controls for a 24 h period. Afterwards, cells were washed 3 times with prewarmed PBS and stained with the LIVE/DEATH^TM^ kit (Invitrogen, Waltham, USA cat: L3224) following the manufacturer instructions; briefly, cells were stained with a solution containing ethidium homodimer-1 4 μM and calcein-AM 2 μM and counter stained with hoechst 8.1 nM for 30 min at room temperature. Finally, cells were washed 3 more times with PBS and the constructs were dismounted from the CellCrown^TM^ system for imaging.

Cells were counted as alive when stained positively for calcein and negatively for ethidium homodimer-1, and dead when stained positively for ethidium homodimer-1 or negatively for calcein. Viability was calculated as the ratio of alive cells over the total number of nuclei. Controls were cultured on 12 well plates under the same conditions, negative control was treated with 70 % ethanol for 5 min and washed before staining.

### Coculture

HaCaT or MRC5 cells were seeded on the respective side of the basement membrane-containing scaffold at a density of 60 x 10^3^ cells/cm^2^ and kept in standard culture conditions for 14 days, with two media changes per week. Then, the construct was fixed with 4 % paraformaldehyde in PBS (Klinipath) for 30 min, washed in PBS and mounted on a glass slide. The construct was permeabilized with Triton X-100 0.5 % for 10 min and stained with Phalloidin iFluor 555 (abcam, cat: ab176756) 1:1000 in PBS containing 1 % BSA during 90 min. Finally, the sample was mounted with a coverslip using PBS as mounting agent to be imaged immediately.

### Confocal microscopy and second harmonics generation

Images for cell viability were obtained with a Stellaris 5 platform (Leica microsystems, Germany) with a HC APO L U-V 40X/0.8 W objective (Leica microsystems, working distance 3.3 mm), lasers diodes: 405 nm, 2 %, 488 nm, 0.82 % and 561 nm, 5 %, and HyD S detectors: 420–483 nm,gain 30, 505–556 nm, gain 10, 588–740 nm, gain 30 respectively. The pinhole was 106.1 μm and images were captured with a *xy* resolution of 1024x1024 pixels and *z* stacks with 2 μm steps.

Visualization of the collagen IV surface and actin filaments together SHG for collagen fibers was done in a Stellaris 8 multiphoton platform (Leica microsystems, Germany) equipped with a pulsed white light laser set at 551 nm 5 % intensity and a CRS Laser picoEmerald S (Ape, Berlin, Germany) set at 1031 nm 0.3 W, 60 % power employing a HC PL IRAPO 40x/1.10 W CORR objective (Leica microsystems), HyD S detector: 556–613 nm, gain 66, F SHG gain 67, E SHG 80 together with CARS 2000S filters and a quarter wave plate. Images were acquired with 1024x1024 pixel *xy* resolution and *z* step of 0.424 μm with a pinhole 77.2 μmϕ.

The acquisition software used in both microscopes was LasX (Leica microsystems, Germany), images were latter processed with Fiji and 3D reconstructions were done with LasX (Leica microsystems, Germany).

### Statistical analysis

The thickness of 14 dissected cellular fresh mucosae was measured independently, and from those, 6 were measured after decellularization (basement membrane-containing scaffolds); 3 random fields were scanned, and 10 measurements were done per field. Five independent bladders obtained on different days were used to test the mechanical properties in the two orthogonal directions, and three for decellularization and stress relaxation, with three replicate samples from each bladder. AFM tests were carried out on 3 independent bladders. For cell viability, 3 independent samples were tested, and at least 3 random fields were captured from each sample. Saphiro Wilk normality tests were conducted for each group, two-way repeated measures ANOVA when comparisons were made within the same bladder or one-way ANOVA was performed otherwise with an α level of 0.05. An ordinary ANOVA test with Tukey’s correction for multiple comparison was performed on the strain relaxation at 5 %, 10 % and 15 % strain. The tests and graphics were made with GraphPad Prism 8 (GraphPad software, Boston, USA), mean and standard deviation of the mean are plotted.

## Funding information

Jhon Ramirez is granted with the Minciencias (Colombian ministry of Science, Technology and Innovation) scholarship call 906 of 2021.

## CRediT authorship contribution statement

**J.A. Ramirez:** Writing – review & editing, Writing – original draft, Visualization, Software, Methodology, Investigation, Formal analysis, Data curation, Conceptualization. **S. Estrada:** Writing – review & editing, Supervision, Methodology, Investigation, Conceptualization. **M.C. Harmsen:** Writing – review & editing, Supervision, Methodology. **P.K. Sharma:** Writing – review & editing, Supervision, Methodology, Funding acquisition, Data curation.

## Declaration of competing interest

The authors declare that they have no known competing financial interests or personal relationships that could have appeared to influence the work reported in this paper.

## Data Availability

Data will be made available on request.
