# Supplementary material for: Development of an in vitro platform for epithelial-stromal interactions: A basement membrane-containing scaffold from decellularized porcine bladders
Source: Matrix Biol Plus. 2025 Feb 22;26:100169. doi: 10.1016/j.mbplus.2025.100169 (PMC11928823; doi:10.1016/j.mbplus.2025.100169)
Supplement: Supplementary Data 4 [file mmc4.docx]

**
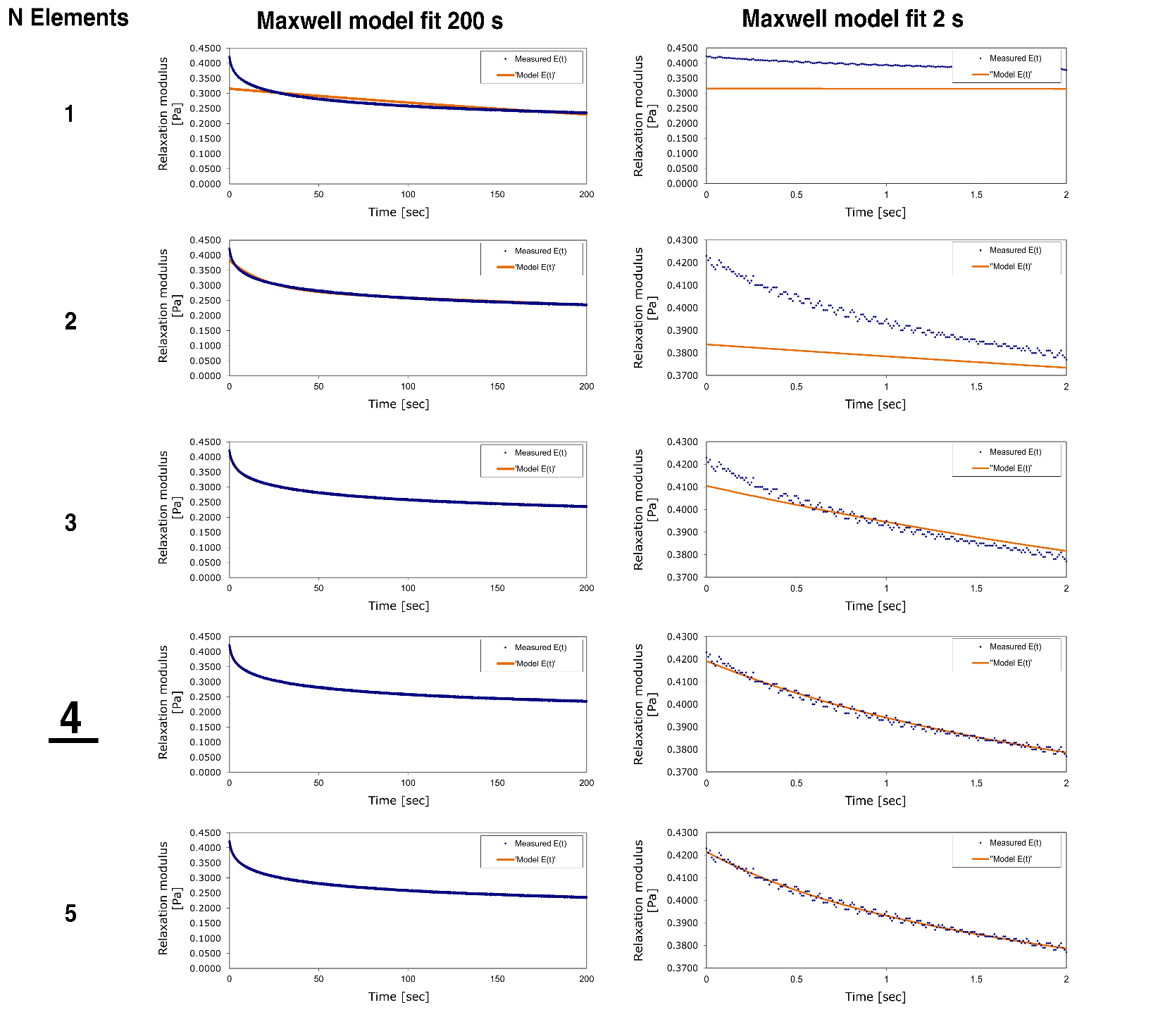
**

**Supplementary figure 1 Fitting of Maxwell elements** from 1 to 5 elements, we choose 4 as the most accurate fit with the minimum number of elements.

**
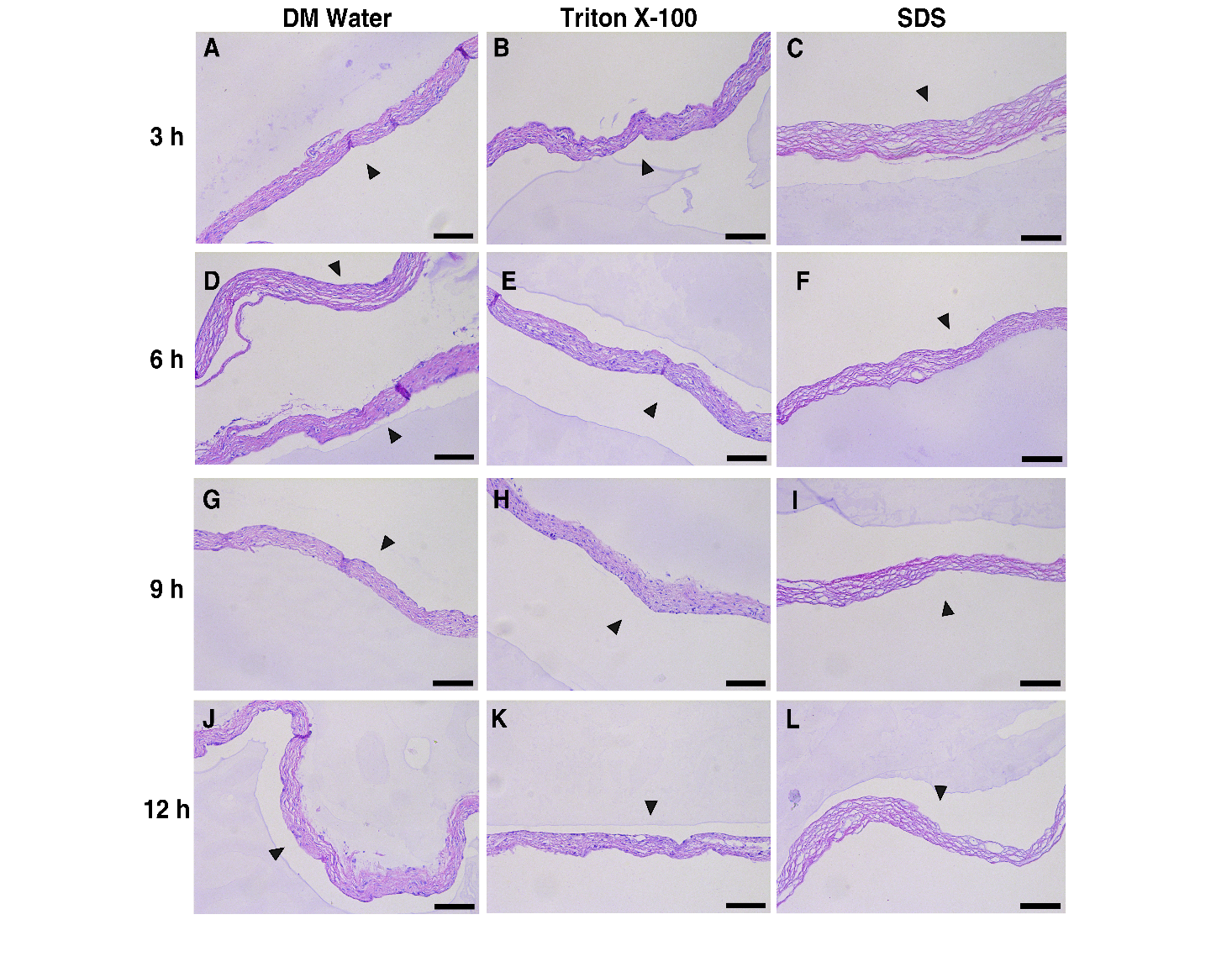
**

**Supplementary Fig 2 Standardization of decellularization methods.** Sections of BM scaffolds treated with DM water (**A**, **D**, **G** and **J**), Triton X-100 (**B**, **E**, **H** and **K**) and SDS (**C**, **F**, **I** and **L**) are shown at different time points to determine the extent of decellularization. Arrowheads indicate the BM surface. Scale bars are 100μm.

**
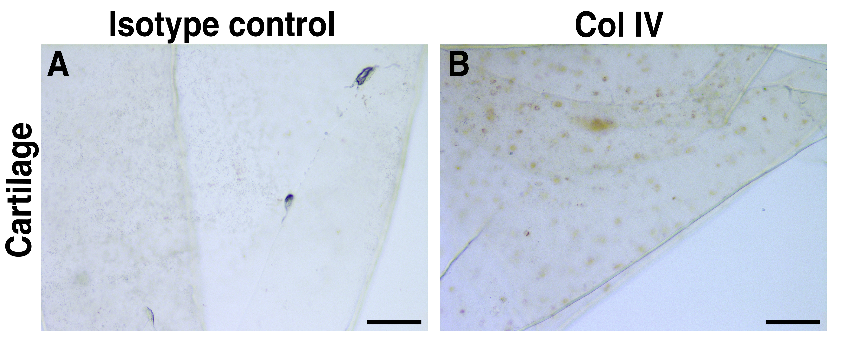
**

**Supplementary Fig 3 collagen IV antibody validation on cartilage A)** section staining without primary antibody (control), **B)** section stained for collagen IV. Scale bars are 100μm.

**
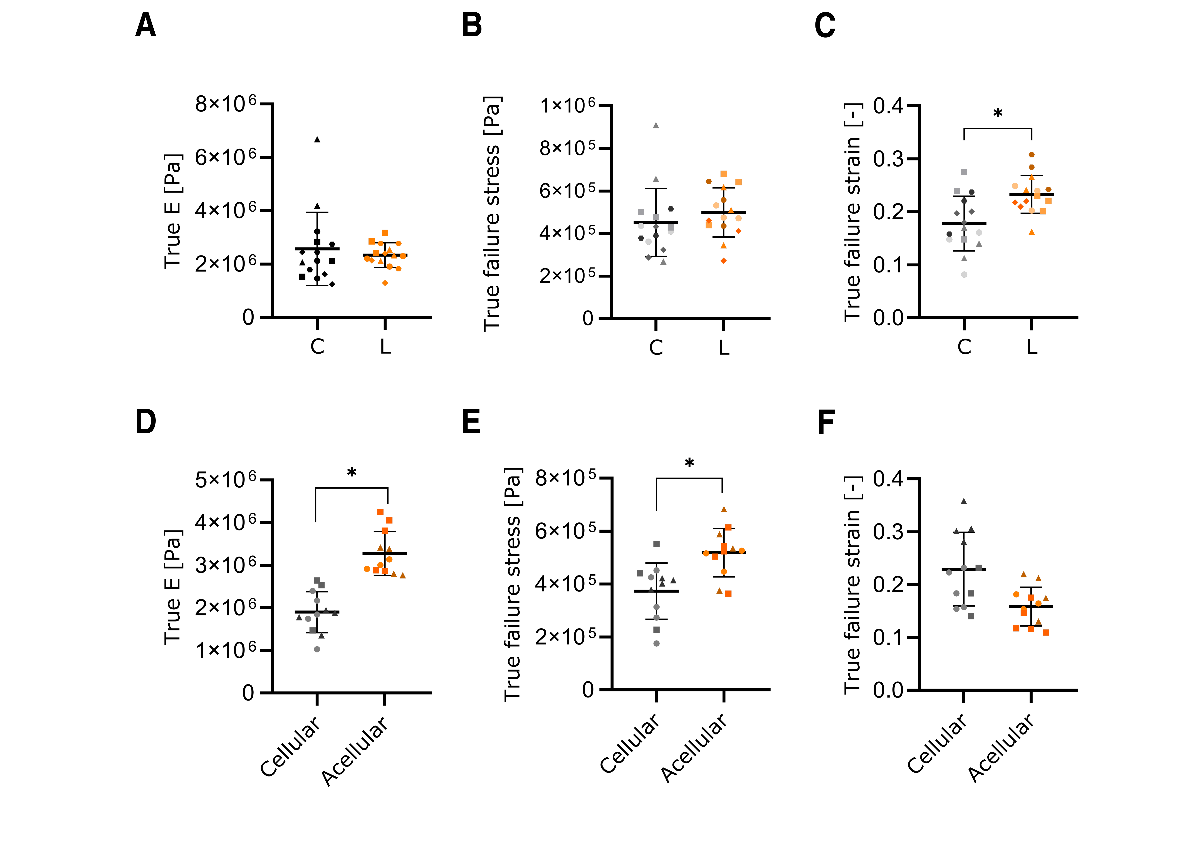
**

**Supplementary Fig 4 True values of Young’s modulus and failure stress and strain** calculated as $\sigma_{t}=\sigma(1-\varepsilon)$, and $\varepsilon_{t}=ln(1+\varepsilon)$ respectively, where $\sigma$ and $\varepsilon$ are the engineering stress and strain.

**
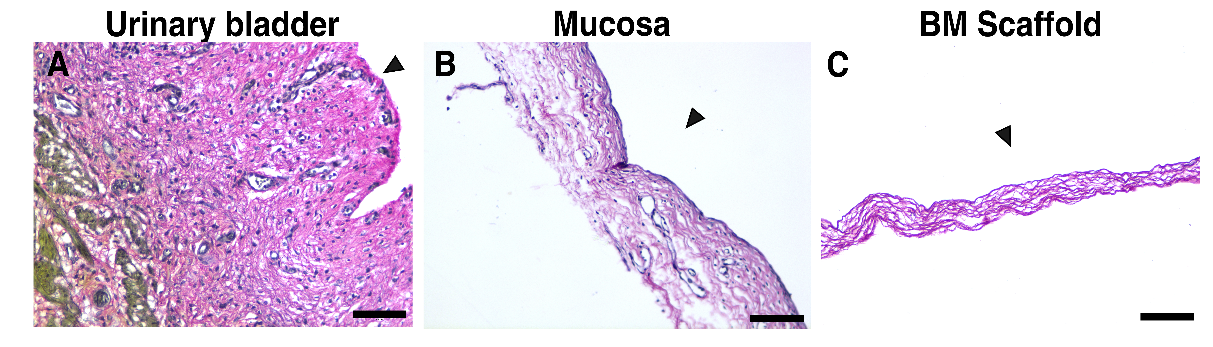
**

**Supplementary Fig 5 Verhoeff’s elastin staining** **A)** Urinary bladder, **B)** Dissected mucosa and **C)** basement membrane-containing scaffold. Arrow heads indicate the BM surface. Scale bars are 100μm
